# Mitochondria hyperactivity contributes to social behavioral impairments

**DOI:** 10.1038/s41392-020-00239-y

**Published:** 2020-07-20

**Authors:** Yun Zhang, Lin Peng, Weihong Song

**Affiliations:** 1 Townsend Family Laboratories, Department of Psychiatry, The University of British Columbia, 2255 Wesbrook Mall, Vancouver, BC V6T 1Z3 Canada; 2 Advanced Innovation Center for Human Brain Protection, National Clinical Research Center for Geriatric Disorders, Xuanwu Hospital, Capital Medical University, Beijing, 100053 China; 3 Shandong Collaborative Innovation Center for Diagnosis, Treatment and Behavioral Interventions of Mental Disorders, Institute of Mental Health, Jining Medical University, Jining, 272000 Shandong China

**Keywords:** Molecular neuroscience, Neurodevelopmental disorders

## Mitochondria and social disorders

In a recent article in cell, Kanellopoulos et al. reported that Aralar, as mitochondrial GABA transporter, mediates the effect of mitochondrial hyperactivity on causing social behavior deficits.^1^

**Fig. 1:**
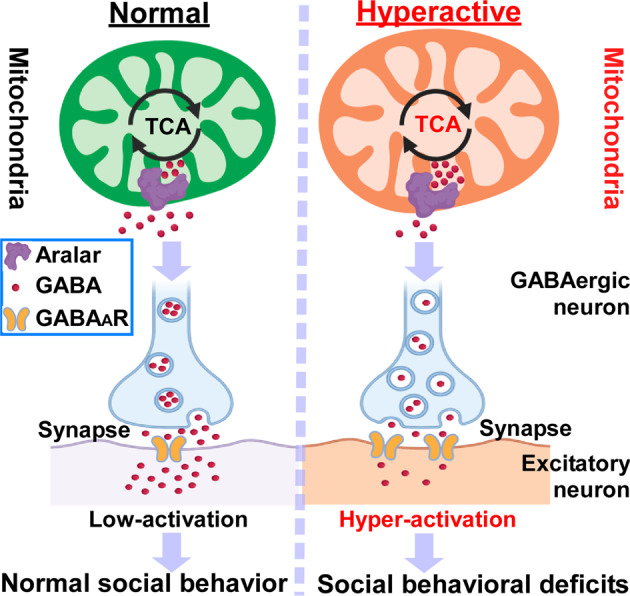
Mitochondrial hyperactivity reduces GABA neurotransmission, leading to social behavioral deficits. CYFIP haploinsuffiency enhances mitochondrial activity by increasing mitochondrion size, membrane potential, and energy metabolism, which further stimulates the mitochondrial translocation of GABA by Aralar carrier. The reduction of GABAergic signaling leads to the social behavioral deficits in neuropsychiatric disorders such as ASD, SCZ, and epilepsy

As the “powerhouse of the cell”, mitochondria provide the majority of energy to neurons and play a key role in the maintenance of brain homeostasis and functionality. Although mitochondrial dysfunction has been observed in social disorders, the exact role of mitochondria and energy metabolism in social behaviors remains unknown. Kanellopoulos and colleagues^1^ discovered that mitochondrial hyperactivity in GABAergic neurons contributes to social behavioral impairments by redistributing GABA neurotransmitters from synaptic compartments to mitochondria via Aralar carrier (Fig. 1). The findings demonstrate that mitochondrial homeostasis is essential for GABA signaling-mediated social behaviors, and provide a new mechanism underlying neuropsychiatric disorders, which opens a potential therapeutic avenue to their treatment.

Social behavioral deficits are a characteristic of many neurodevelopmental and psychiatric disorders, including autism spectrum disorder (ASD), schizophrenia (SCZ), epilepsy, and major depressive disorder (MDD). Common genetic variants such as variants in cytoplasmic FMR1-interacting protein 1 (CYFIP1) gene have been associated with ASD and SCZ. Kanellopoulos et al., found that Cyfip^85.1^/+ flies with CYFIP haploinsufficiency displayed ASD-like and SCZ-like behavioral impairments. By performing proteomic analysis, mitochondrial metabolism was further recognized as the key molecular mechanism underlying these behavioral alterations observed in Cyfip^85.1^/+ flies.

Mitochondria are double-membrane-bound organelles that produce large quantities of energy in the form of adenosine triphosphate (ATP) via oxidative phosphorylation (OXPHOS), and are as such pivotal for cell survival and function. As a high energy-consuming tissue, the brain is greatly dependent on mitochondria for its development and functionality. Mitochondria play a key role in the fundamental processes of neuroplasticity including neural differentiation, outgrowth of axons and dendrites, synapse formation, neurotransmitter release, and dendritic remodeling. In addition, mitochondria keep pace with their metabolic environment by changing their morphology and activity, which may also influence the neuronal activity and brain function. This new report shows that CYFIP deficiency induced the morphological changes of mitochondria with enlarged area and perimeter, increased mitochondrial membrane potential and stimulated glycolysis to increase TCA/Krebs cycle, resulting in mitochondrial hyperactivity. Reduction of mitochondrial activity by inhibition of IDH enzyme successfully ameliorated the abnormal social behaviors in Cyfip^85.1^/+ flies, indicating that mitochondrial hyperactivity plays a causal role in social behavioral impairment due to CYFIP insufficiency.

To determine whether a specific cell type is responsible for CYFIP’s effect on social behaviors in the flies, the authors knocked down CYFIP in both excitatory (cholinergic) and inhibitory (GABAergic) neurons. The results showed that the behavioral changes only occurred in Cyfip^85.1^/+ flies with depletion of CYFIP in anterior paired lateral or antennal lobe local GABAergic neurons, and reduction of Cyfip mRNA expressions was specifically found in the GABAergic neurons. Gamma aminobutyric acid (GABA), the main inhibitory neurotransmitter, is synthesized from the excitatory neurotransmitter glutamate via the action of glutamate decarboxylase (GAD) enzymes, including GAD65 and GAD67. During perinatal, GABA induces the depolarization of target cells to trigger calcium influx. GABA-mediated calcium signaling plays an important role in cell proliferation, differentiation, and death as well as synapse maturation. Impairment of the GABAergic signaling leads to an excitatory/inhibitory imbalance in neuronal circuits, which accounts for social behavioral deficits observed in patients with ASD or SCZ. In contrast, enhancement of GABA signaling ameliorates social dysfunction in both ASD and epilepsy model mice.^2^ Consistent with these studies, Kanellopoulos et al. found that CYFIP insufficiency reduced GABA neurotransmission by translocation of GABA into mitochondria. The elevated GABA levels in mitochondria further stimulate NADH and succinate generation for TCA /Krebs cycle, leading to increased mitochondrial activity. In contrast, augmentation of GABA levels improved the behavioral deficits in the flies. These findings suggest that insufficient vesicle GABA due to sequestering GABA to mitochondria contributes to CYFIP-induced social deficits.

The mitochondrial carrier system transports molecules between mitochondria and the cytoplasm. In this report, the authors identified that Aralar is the novel transporter protein for shuttling GABA into the mitochondria in *Drosophila*. Aralar is a member of the SLC25 family located on the inner mitochondrial membrane to facilitate the transport of solutes. It is mainly expressed in brain and skeletal muscle. Aralar plays a key role in the exchange of cytoplasmic glutamate with mitochondrial aspartate in a calcium-dependent manner. As a member of the malate-aspartate NADH shuttle, Aralar is also involved in the transfer of electrons from the cytosol to the mitochondrial matrix for oxidative phosphorylation.^3^ Two single nucleotide polymorphisms (SNPs) in Aralar-encoding gene *SLC25A12* have been identified to associate with ASD^4^ and *SLC25A12* expression is upregulated in ASD patients.^5^ Aralar transporter activity was upregulated by the reduction of CYFIP in *cyfip*^85.1^/+ flies due to enhanced mitochondrial metabolism and membrane potential. Inhibition of Aralar’s activity to transfer GABA into mitochondria by pyridoxal 5′-phosphate (PLP) or *Aralar*^M107552^ mutation resulted in the significant improvement of behavioral deficits in *cyfip*^85.1^/+ flies. These findings unveil the important role of Aralar in linking mitochondrial activity, GABA transmission, and social function.

There are currently no effective the Food and Drug Administration (FDA)-approved medications to treat the core social and communicative impairments of disorders, such as ASD. This is the first study to demonstrate the causal role of brain mitochondrial hyperactivity in social behavioral impairment and uncover the underlying mechanism. The findings shed a new light in the development of novel strategies to treat these social disorders. The authors found that increased cytosolic GABA levels by mutation of *Aralar* also cause social behavioral deficits similar to *Cyfip* mutation, indicating that both deficiency and excess of GABA neurotransmitters are detrimental. Therefore, targeting mitochondrial activity rather than GABA or Aralar per se seems to be a more promising approach to improve social deficits. Further studies could reveal the underlying mechanism by which CYFIP induces mitochondrial hyperactivity and validate the Aralar–GABA’s effect in rodent models. Reactive oxygen species (ROS) have been shown to regulate GABAergic signaling and increased ROS levels and oxidative stress are observed in ASD and SCZ. It would be interesting to see whether ROS also plays a role in social behavioral impairment induced by mitochondria hyperactivity.

## References

[1] Kanellopoulos AK (2020). Aralar sequesters GABA into hyperactive mitochondria, causing social behavior deficits. Cell.

[2] Han S, Tai C, Jones CJ, Scheuer T, Catterall WA (2014). Enhancement of inhibitory neurotransmission by GABAA receptors having alpha2,3-subunits ameliorates behavioral deficits in a mouse model of autism. Neuron.

[3] Satrustegui J (2007). Role of aralar, the mitochondrial transporter of aspartate-glutamate, in brain N-acetylaspartate formation and Ca(2+) signaling in neuronal mitochondria. J. Neurosci. Res..

[4] Segurado R (2005). Confirmation of association between autism and the mitochondrial aspartate/glutamate carrier SLC25A12 gene on chromosome 2q31. Am. J. Psychiatry.

[5] Lepagnol-Bestel AM (2008). SLC25A12 expression is associated with neurite outgrowth and is upregulated in the prefrontal cortex of autistic subjects. Mol. Psychiatry.

